# Crystal structure of 3-meth­oxy-4-[2-(thia­zol-2-yl)diazen-1-yl]aniline monohydrate

**DOI:** 10.1107/S205698901900207X

**Published:** 2019-02-12

**Authors:** Nutchanikan Phiromphu, Kittipong Chainok, Apisit Songsasen, Tanwawan Duangthongyou

**Affiliations:** a Department of Chemistry, Faculty of Science, Kasetsart University, Bangkok, 10900, Thailand; b Materials and Textile Technology, Faculty of Science and Technology, Thammasat, University, PathumThani 12120, Thailand

**Keywords:** crystal structure, azo dye, thia­zole ring, hydrogen bonding, C—H⋯π inter­action

## Abstract

In the title hydrated azo dye, C_10_H_10_N_4_OS·H_2_O, the benzene and thia­zole, are nearly coplanar, with a dihedral angle between their mean planes of 4.69 (17)°. The aromatic rings on the –N=N– moiety exhibit a *trans* configuration. The crystal structure features many types of inter­molecular inter­actions involving all the functional groups – strong hydrogen bonds (N⋯H and O⋯H), weak hydrogen bonds (C—H⋯O and C—H⋯N), C—H⋯π and π–π inter­actions – resulting in the formation of a three-dimensional framework.

## Abstract 2

In the title hydrated azo dye, the benzene and thia­zole make a dihedral angle of 4.69 (17)°. In the crystal, hydrogen bonds, C—H⋯π and π–π inter­actions resulting in the formation of a three-dimensional framework.

## Chemical context   

Thia­zolylazo compounds contain a thia­zole ring and an azo group (–N=N–). Azo dyes have wide range applications in the cosmetic, food, textile industry, chemical sensing, and pharmaceutical (Weglarz-Tomczak & Gorecki, 2012 ▸) fields. 4-(2-Thia­zolylazo) resorcinol (TAR) was the first thia­zolylazo dye (Jensen, 1960 ▸). Changing the substituent groups on the azo bond (Hovind, 1975 ▸) changes the coordination properties with metal ions, as in the complexation of 1-(2-thia­zolylazo)-2-naphthol (TAN) with transition metals (Omar *et al.*, 2005 ▸). Cleavage of the azo bond occurs in reductive metabolism of mammalian systems (Levine, 1991 ▸) that can decrease or increase any toxic or carcinogenic effects of the dyes. Sutthivaiyakit *et al.* (1998 ▸) described the preparation of a new chelating silica with 2-(2-thia­zolylazo)-5-amino­anisole used for a stationary phase in high-pressure liquid chromatography. In this work, we report the structure of 3-meth­oxy-4-[2-(thia­zol-2-yl)diazen-1-yl]aniline monohydrate, also known as 2-(2-thia­zolylazo)-5-amino­anisole (*p*-amino TAA), (I). Future work will study its complexation with metal ions.
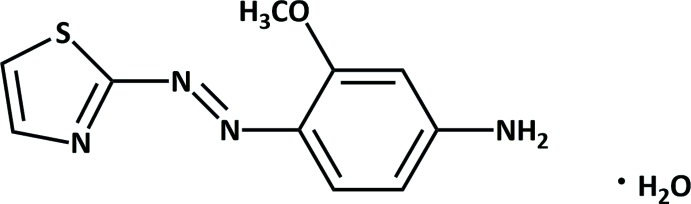


## Structural commentary   

**Figure 1:**
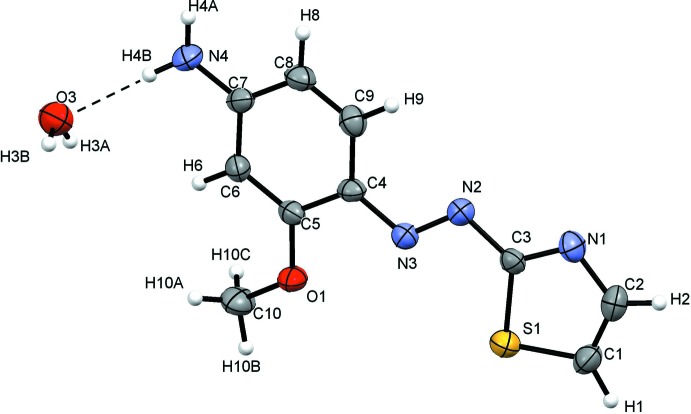
The mol­ecular structure of compound (I) with the atom labelling and 50% probability displacement ellipsoids

The mol­ecular structure of (I) is shown in Fig. 1 ▸. The thia­zole and benzene rings are arranged *trans* to the azo bridge (–N2=N3–). The meth­oxy and amino groups on the benzene ring are co-planar with the ring with atoms O1 and N4 deviating by −0.010 (2) and −0.019 (4) Å, respectively. The dihedral angle between the thia­zole and benzene rings is 4.69 (17)°, nearly coplanar.

## Supra­molecular features   

**Figure 2:**
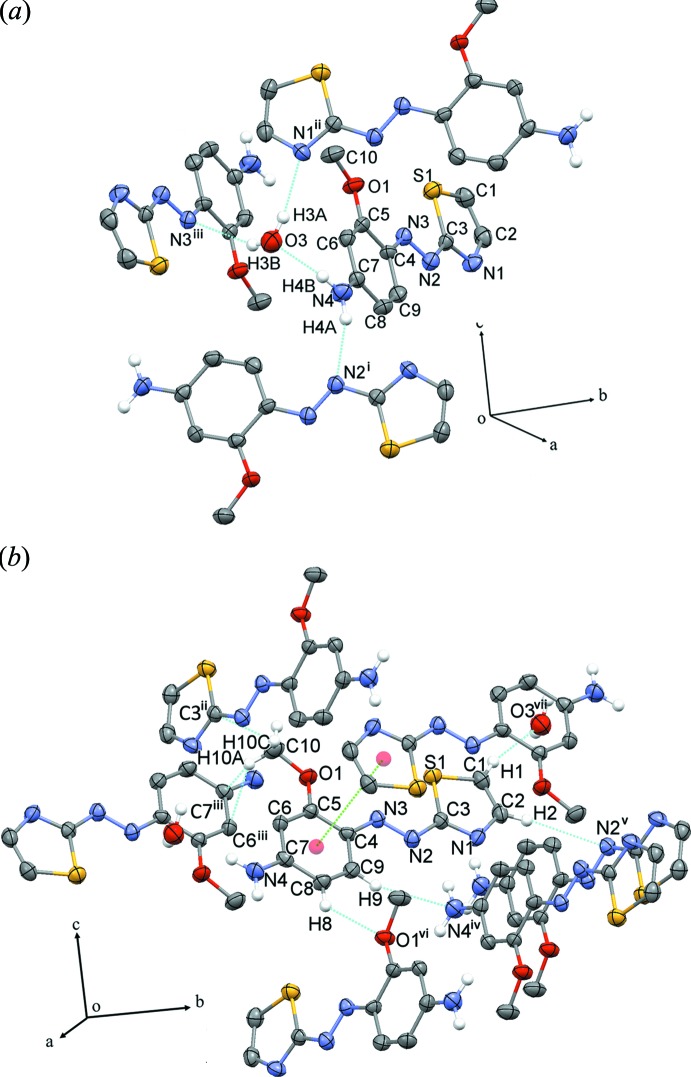
(*a*) The packing of the crystal by strong hydrogen bonds and (*b*) secondary inter­actions. Symmetry codes: (i) −*x* + 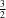, *y* − 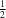, −*z* + 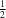; (ii) *x* + 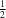, −*y* + 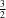, *z* + 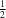; (iii) −*x* + 1, −*y* + 1, −*z* + 1; (iv) −*x* + 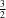, *y* + 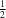, −*z* + 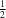; (v) −*x* + 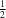, *y* + 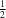, −*z* + 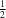; (vi) *x* + 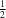, −*y* + 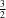, *z* − 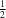; (vii) *x* − 1, *y* + 1, *z*.

In the crystal, three-dimensional structure is generated by contribution of strong and weak hydrogen bonding, C—H⋯π inter­actions and offset π–π inter­action. The strong hydrogen bonds (Fig. 2 ▸ *a*, Table 1 ▸), which involve the amine (NH_2_), azo (–N=N–) and thia­zole groups and the water mol­ecule of crystallization [N4—H4*B*⋯O3, O3—H3*A*⋯N1^ii^, O3—H3*B*⋯N3^iii^, N4—H4*A*⋯N2^i^] are the primary inter­actions responsible for the formation of the three dimensional structure. In addition, the crystal structure is supported by other inter­molecular inter­actions as a secondary weak inter­actions, C—H⋯*X* (*X =* O and N), C—H⋯π and offset π—π inter­actions. The weak hydrogen bonds are formed between the C—H moieties in the benzene and thia­zole rings with amine, azo, meth­oxy groups of adjacent mol­ecules and water mol­ecules [C1—H1⋯O3^vii^, C2—H2⋯N2^v^, C8—H8⋯O1^vi^ and C9—H9⋯N4^iv^. The C—H⋯π inter­actions involve the meth­oxy group and ring carbon atoms [C10—H10*C*⋯C3^ii^, C10—H10*A*⋯C6^iii^ and C10—H10*A*⋯C7^iii^ while the offset π–π inter­action is formed between benzene and thia­zole rings with a centroid–centroid distance of 3.850 (5) Å, symmetry operation 1 − *x*, 2 − *y*, 1 − *z* (Fig. 2 ▸ *b*, Table 1 ▸).

## Hirshfeld surface analysis   

**Figure 3:**
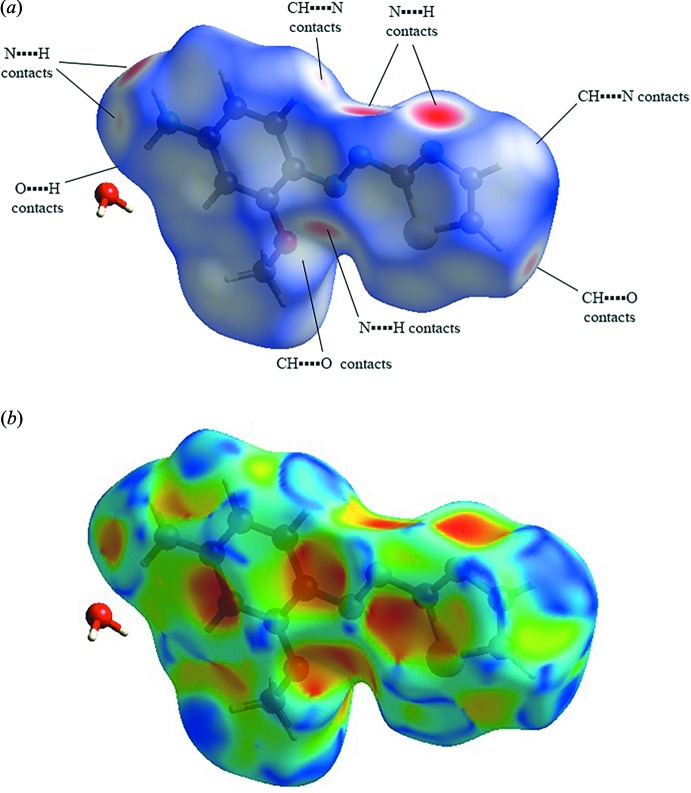
Hirshfeld surfaces for compound (I), mapped with (*a*) *d*_norm_ and (*b*) shape-index.

Hirshfeld surfaces and fingerprint plots were generated using *CrystalExplorer* (Hirshfeld, 1977 ▸; McKinnon *et al.*, 2004 ▸). Fig. 3 ▸ shows the Hirshfeld surface of compound (I) mapped over *d*_norm_ (−0.5129 to 1.1405 Å) and the shape index (−1.0 to 1.0 Å). The red spots in the Hirshfeld surface represent short N⋯H and O⋯H contacts and correspond to hydrogen-bonding inter­actions between (NH_2_)N—H⋯N(azo), (H_2_O)O—H⋯N(azo), (H_2_O)O—H⋯N(thia­zole) and (NH_2_)N—H⋯O(H_2_O). The pale-red spots result from the weak C—H⋯O(H_2_O) and C—H⋯N(NH_2_) hydrogen-bonding inter­actions. The white spots in Fig.3*a* represent long contacts [C—H⋯N(azo) and C—H⋯O(OCH_3_)]. On the shape index surface (Fig. 3 ▸ *b*), convex blue regions represent hydrogen-donor groups and concave red regions represent hydrogen-acceptor groups. In addition, concave red regions represent C—H⋯π and offset π–π inter­actions. The amino group behaves as both a donor and an acceptor. The methyl part of the meth­oxy group acts as a donor while the oxygen atom is an acceptor.

**Figure 4:**
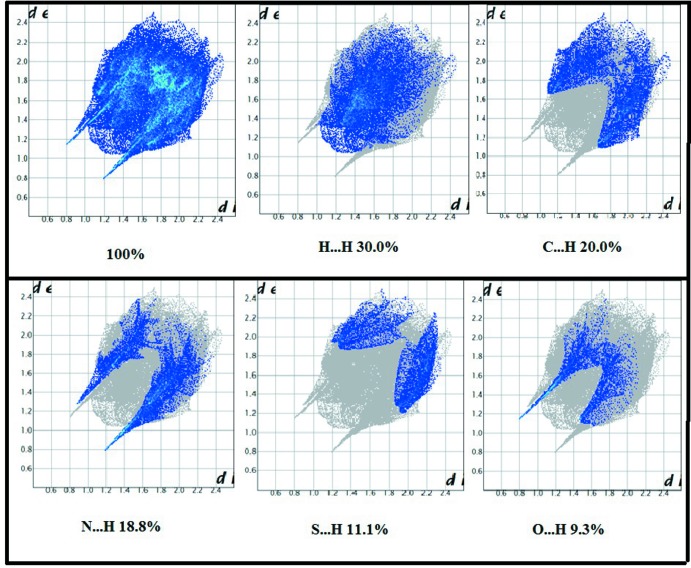
Two-dimensional fingerprints for compound (I), showing H⋯H, C⋯H, N⋯H, S⋯H and O⋯H contacts.

The two-dimensional fingerprint plots (Fig. 4 ▸) qu­antify the contributions of each type of inter­molecular inter­action to the Hirshfeld surface (McKinnon *et al.*, 2007 ▸). The largest contribution with 30.0% of the surface is from H⋯H contacts, which represent van der Waals inter­actions, followed by C⋯H contacts involved in C—H⋯π inter­actions (20.0%). In the N⋯H plot (18.8% contribution), the two sharp peaks correspond to strong hydrogen bonds. Finally, the O⋯H (9.3%), S⋯H (11.1%) and C⋯C (3.3%) contacts correspond to hydrogen bonds and offset π–π inter­actions, respectively.

## Database survey   

Related compounds to (I) are substituted thia­zolylazo derivatives, for example 4-(2-thia­zolylazo) resorcinol (TAR), 1-(2-thia­zolylazo)-2-naphthol (TAN) and 2-(2-thia­zolylazo)-4-methyl­phenol (TAC) (Jensen, 1960 ▸). These thia­zolylazo derivatives are used as chelating agents with metal ions (Farias *et al.*, 1992 ▸). In the crystal structure of 1-(2-thia­zolylazo)-2-naphthol (TAN; Kurahashi, 1976 ▸), the azo group adopts a *trans* configuration and the phenolic oxygen atom is linked to an azo nitro­gen atom by intra­molecular hydrogen bonding. The crystal structure features only van der Waals inter­actions. To form complexes with metal ions, both thia­zole and naphthol rings are rotated by 180° to coordinate to the metal through the phenolic oxygen atom, the azo nitro­gen atom adjacent to the naphthol ring and the thia­zole nitro­gen atom, resulting the formation of five-membered chelate rings. Complexes of TAR and TAC are formed in a similar way due to the presence of a hydroxyl group in the structure (Karipcin *et al.*, 2010 ▸). 3-[2-(1,3-Thia­zol-2-yl)diazen-1-yl]pyridine-2,6-di­amine monohydrate (Chotima *et al.*, 2018 ▸) has been used as a chelating ligand to form a complex with Au^III^ ion (Piyasaengthong *et al.*, 2015 ▸). The crystal structure is stabilized by hydrogen bonding between the amine group, water and the thia­zole nitro­gen atom along with π–π inter­actions between pairs of pyridine rings and pairs of thia­zole rings, resulting in the formation of a layered structure. In addition, weak C—H⋯S hydrogen bonds between adjacent thia­zole rings further contribute to the crystal packing, generating a three-dimensional network.

## Synthesis and crystallization   

2-Amino­thia­zole (9.986 mmol) was dissolved in 6 *M* HCl (16 ml), and 8.236 mmol of sodium nitrate solution was added slowly under stirring at low temperature 268–273 K until the diazo­nium salt was obtained. *m*-Anisidine (1.12 ml in 40 ml of 4 *M* HCl) was slowly dropped into the mixture and stirred at a temperature between 268 and 273 K for 1 h. After the reaction was complete, conc. NH_3_ was dropped into the mixture (pH 6) until the red–orange crude produce appeared. The products were filtered, washed with cold water, purified by column chromatography and recrystallized from an aceto­nitrile–water (1:1) mixture by vapour diffusion.

^1^H NMR (400 MHz, DMSO-*d*_6_): δ 3.806 (3H, *s*, H^c^), 6.364 (1H, *dd*, H^f^, *J* = 8.7, 2.7 Hz) , 6.374 (1H, *t*, H^d^, *J* = 2.8 Hz), 7.546 (1H, *d*, H^g^, *J* = 8.9 Hz) ,7.629 (1H, *d*, H^a^, *J* = 3.40 Hz), 7.697 (2H, *s*, H^e^), 7.883 (1H, *d*, H^b^, *J* = 3.42 Hz). Mass spectroscopy: *m*/*z* 235.0654 [C_10_H_11_N_4_OS^+^], 205.0548 [C_9_H_9_N_4_S^.+^], 150.0662 [C_7_H_8_N_3_O^.+^], 122.0601 [C_7_H_8_NO^.+^]. IR (KBr cm^−1^): 3,413 cm^−1^ (*s*, N—H); 821 cm^−1^ (*w*, NH_2_); 1,617 (*m*, C=N); 1,222 cm^−1^ (*w*, C—N stretch aromatic amine); 1,103 cm^−1^ (*m*, C—N stretch amine); 1,152 cm^−1^ (*m*, C—S); 1,541cm^−1^ (*m*, N=N); 1,021 cm^−1^ (*w*, C—O stretch). Elemental analysis calculated for C_10_H_10_N_4_OS·H_2_O: C, 51.27; H, 4.30; N, 23.92. Found: C, 51.34; H, 4.20; N, 23.98.

## Refinement   

Crystal data, data collection and structure refinement details are summarized in Table 2 ▸. Water and amino H atoms were refined freely while those of aromatic and methyl groups were placed in calculated positions (C—H = 0.93 and 0.96 Å, respectively) and included in the cycles of refinement using a riding model with *U*_iso_ = 1.2 *U*_eq_(C-aromatic) and 1.5*U*_eq_ (C-meth­yl).

## Supplementary Material

Crystal structure: contains datablock(s) global, I. DOI: 10.1107/S205698901900207X/dx2014sup1.cif

Structure factors: contains datablock(s) I. DOI: 10.1107/S205698901900207X/dx2014Isup2.hkl

CCDC reference: 1895710

Additional supporting information:  crystallographic information; 3D view; checkCIF report

## Figures and Tables

**Table 1:** Hydrogen-bond geometry (Å, °)

*D*—H⋯*A*	*D*—H	H⋯*A*	*D*⋯*A*	*D*—H⋯*A*
N4—H4*A*⋯N2^i^	0.87 (1)	2.30 (2)	3.137 (5)	162 (3)
N4—H4*B*⋯O3	0.87 (1)	2.08 (1)	2.946 (5)	173 (4)
O3—H3*A*⋯N1^ii^	0.84 (1)	2.12 (2)	2.954 (5)	169 (6)
O3—H3*B*⋯N3^iii^	0.84 (1)	2.43 (3)	3.186 (5)	150 (5)
C9—H9⋯N4^iv^	0.93	2.69	3.587 (5)	162
C2—H2⋯N2^v^	0.93	2.87	3.798 (5)	176
C8—H8⋯O1^vi^	0.93	2.72	3.452 (5)	136
C1—H1⋯O3^vii^	0.93	2.56	3.463 (5)	165
C10—H10*C*⋯C3^ii^	0.96	2.89	3.655 (5)	137
C10—H10*A*⋯C6^iii^	0.96	2.83	3.551 (5)	132
C10—H10*A*⋯C7^iii^	0.96	2.86	3.502 (5)	125

Symmetry codes: (i) 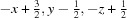; (ii) 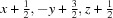; (iii) 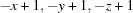; (iv) 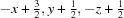; (v) 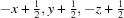; (vi) 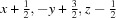; (vii) 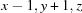.

**Table 2:** Experimental details

Crystal data	
Chemical formula	C_10_H_10_N_4_OS·H_2_O
*M*_r_	252.30
Crystal system, space group	Monoclinic, *P*2_1_/*n*
Temperature (K)	298
*a*, *b*, *c* (Å)	9.051 (5), 11.526 (5), 10.893 (6)
β (°)	90.345 (16)
*V* (Å^3^)	1136.5 (10)
*Z*	4
Radiation type	Mo *K*α
μ (mm^−1^)	0.28
Crystal size (mm)	0.14 × 0.06 × 0.06

Data collection	
Diffractometer	Bruker APEXII CCD
Absorption correction	Multi-scan (*SADABS*; Bruker, 2016 ▸)
*T*_min_, *T*_max_	0.585, 0.745
No. of measured, independent and observed [*I* > 2σ(*I*)] reflections	13093, 2164, 995
*R*_int_	0.164
(sin θ/λ)_max_ (Å^−1^)	0.611

Refinement	
*R*[*F* ^2^ > 2σ(*F* ^2^)], *wR*(*F* ^2^), *S*	0.055, 0.131, 0.93
No. of reflections	2164
No. of parameters	172
No. of restraints	4
H-atom treatment	H atoms treated by a mixture of independent and constrained refinement
Δρ_max_, Δρ_min_ (e Å^−3^)	0.26, −0.26

Computer programs: *APEX3* and *SAINT* (Bruker, 2016 ▸), *SHELXT* (Sheldrick, 2015*a* ▸), *SHELXL2016* (Sheldrick, 2015*b* ▸) and *OLEX2* (Dolomanov *et al.*, 2009 ▸).

## supplementary crystallographic information

### Crystal data

**Table:**

C_10_H_10_N_4_OS·H_2_O	*F*(000) = 528
*M**_r_* = 252.30	*D*_x_ = 1.475 Mg m^−^^3^
Monoclinic, *P*2_1_/*n*	Mo *K*α radiation, λ = 0.71073 Å
*a* = 9.051 (5) Å	Cell parameters from 395 reflections
*b* = 11.526 (5) Å	θ = 2.9–19.0°
*c* = 10.893 (6) Å	µ = 0.28 mm^−^^1^
β = 90.345 (16)°	*T* = 298 K
*V* = 1136.5 (10) Å^3^	Block, brown
*Z* = 4	0.14 × 0.06 × 0.06 mm

### Data collection

**Table:**

Bruker APEXII CCD diffractometer	2164 independent reflections
Radiation source: microfocus sealed X-ray tube, Incoatec Iµs	995 reflections with *I* > 2σ(*I*)
Mirror optics monochromator	*R*_int_ = 0.164
Detector resolution: 7.9 pixels mm^-1^	θ_max_ = 25.8°, θ_min_ = 2.6°
φ and ω scans	*h* = −10→11
Absorption correction: multi-scan (SADABS; Bruker, 2016)	*k* = −14→12
*T*_min_ = 0.585, *T*_max_ = 0.745	*l* = −13→13
13093 measured reflections	

### Refinement

**Table:**

Refinement on *F*^2^	Hydrogen site location: mixed
Least-squares matrix: full	H atoms treated by a mixture of independent and constrained refinement
*R*[*F*^2^ > 2σ(*F*^2^)] = 0.055	*w* = 1/[σ^2^(*F*_o_^2^) + (0.0242*P*)^2^] where *P* = (*F*_o_^2^ + 2*F*_c_^2^)/3
*wR*(*F*^2^) = 0.131	(Δ/σ)_max_ < 0.001
*S* = 0.93	Δρ_max_ = 0.26 e Å^−^^3^
2164 reflections	Δρ_min_ = −0.25 e Å^−^^3^
172 parameters	Extinction correction: SHELXL2016 (Sheldrick, 2015b), Fc^*^=kFc[1+0.001xFc^2^λ^3^/sin(2θ)]^-1/4^
4 restraints	Extinction coefficient: 0.009 (2)
Primary atom site location: dual	

### Special details

**Table:**

Geometry. All esds (except the esd in the dihedral angle between two l.s. planes) are estimated using the full covariance matrix. The cell esds are taken into account individually in the estimation of esds in distances, angles and torsion angles; correlations between esds in cell parameters are only used when they are defined by crystal symmetry. An approximate (isotropic) treatment of cell esds is used for estimating esds involving l.s. planes.

### Fractional atomic coordinates and isotropic or equivalent isotropic displacement parameters (Å^2^)

**Table:**

	*x*	*y*	*z*	*U*_iso_*/*U*_eq_	
S1	0.22221 (11)	1.00037 (9)	0.49942 (9)	0.0393 (4)	
O1	0.4757 (3)	0.6793 (2)	0.5996 (2)	0.0379 (7)	
O3	0.8582 (4)	0.3028 (3)	0.5520 (4)	0.0512 (9)	
N1	0.3051 (4)	1.0873 (3)	0.2927 (3)	0.0374 (9)	
N2	0.4373 (3)	0.9181 (3)	0.3421 (3)	0.0324 (8)	
N3	0.4436 (3)	0.8375 (3)	0.4254 (3)	0.0297 (8)	
N4	0.8772 (4)	0.5020 (3)	0.3833 (4)	0.0421 (9)	
C1	0.1384 (4)	1.1242 (3)	0.4478 (4)	0.0387 (11)	
H1	0.063837	1.163516	0.488936	0.046*	
C2	0.1954 (4)	1.1567 (3)	0.3399 (4)	0.0393 (11)	
H2	0.162460	1.222749	0.298950	0.047*	
C3	0.3298 (4)	1.0010 (3)	0.3686 (3)	0.0280 (9)	
C4	0.5502 (4)	0.7542 (3)	0.4088 (3)	0.0283 (10)	
C5	0.5698 (4)	0.6708 (3)	0.5038 (3)	0.0285 (9)	
C6	0.6797 (4)	0.5880 (3)	0.4951 (3)	0.0313 (10)	
H6	0.692487	0.534501	0.558164	0.038*	
C7	0.7720 (4)	0.5838 (3)	0.3925 (4)	0.0304 (10)	
C8	0.7527 (4)	0.6669 (3)	0.2975 (3)	0.0364 (11)	
H8	0.813766	0.665287	0.229219	0.044*	
C9	0.6462 (4)	0.7479 (3)	0.3060 (3)	0.0347 (10)	
H9	0.634945	0.801384	0.242785	0.042*	
C10	0.5045 (4)	0.6082 (3)	0.7052 (3)	0.0448 (12)	
H10A	0.495963	0.527893	0.682871	0.067*	
H10B	0.434175	0.625874	0.768158	0.067*	
H10C	0.602526	0.623236	0.735301	0.067*	
H4A	0.927 (4)	0.496 (3)	0.316 (2)	0.056 (14)*	
H4B	0.877 (5)	0.446 (3)	0.437 (3)	0.078 (18)*	
H3A	0.853 (7)	0.340 (5)	0.618 (3)	0.15 (3)*	
H3B	0.776 (3)	0.276 (4)	0.530 (4)	0.10 (2)*	

### Atomic displacement parameters (Å^2^)

**Table:**

	*U*^11^	*U*^22^	*U*^33^	*U*^12^	*U*^13^	*U*^23^
S1	0.0428 (7)	0.0371 (6)	0.0381 (7)	0.0005 (6)	0.0105 (5)	0.0034 (6)
O1	0.0423 (18)	0.0405 (16)	0.0312 (18)	0.0105 (14)	0.0138 (14)	0.0063 (14)
O3	0.053 (3)	0.0464 (19)	0.055 (2)	−0.0033 (18)	0.010 (2)	−0.0018 (18)
N1	0.043 (2)	0.036 (2)	0.033 (2)	0.0055 (18)	0.0006 (17)	0.0102 (18)
N2	0.030 (2)	0.0297 (19)	0.037 (2)	0.0021 (16)	0.0040 (16)	0.0022 (17)
N3	0.028 (2)	0.0277 (18)	0.034 (2)	−0.0010 (16)	0.0017 (15)	0.0006 (17)
N4	0.045 (2)	0.038 (2)	0.044 (3)	0.010 (2)	0.016 (2)	0.000 (2)
C1	0.036 (3)	0.034 (2)	0.046 (3)	0.007 (2)	0.003 (2)	−0.005 (2)
C2	0.045 (3)	0.028 (2)	0.045 (3)	0.006 (2)	−0.005 (2)	−0.001 (2)
C3	0.026 (2)	0.026 (2)	0.032 (2)	−0.005 (2)	0.0027 (18)	0.000 (2)
C4	0.029 (2)	0.031 (2)	0.026 (3)	−0.002 (2)	0.008 (2)	−0.004 (2)
C5	0.031 (2)	0.028 (2)	0.027 (2)	−0.006 (2)	0.0057 (19)	−0.001 (2)
C6	0.037 (3)	0.025 (2)	0.032 (3)	0.001 (2)	0.004 (2)	0.0031 (19)
C7	0.027 (2)	0.029 (2)	0.035 (3)	−0.003 (2)	0.006 (2)	−0.006 (2)
C8	0.038 (3)	0.040 (2)	0.031 (3)	−0.003 (2)	0.010 (2)	0.002 (2)
C9	0.041 (3)	0.033 (2)	0.030 (3)	−0.001 (2)	−0.002 (2)	0.0031 (19)
C10	0.049 (3)	0.055 (3)	0.031 (3)	0.006 (2)	0.011 (2)	0.009 (2)

### Geometric parameters (Å, º)

**Table:**

S1—C1	1.710 (4)	C1—C2	1.340 (5)
S1—C3	1.731 (4)	C2—H2	0.9300
O1—C5	1.355 (4)	C4—C5	1.422 (5)
O1—C10	1.435 (4)	C4—C9	1.423 (5)
O3—H3A	0.842 (10)	C5—C6	1.382 (5)
O3—H3B	0.840 (10)	C6—H6	0.9300
N1—C2	1.377 (5)	C6—C7	1.401 (5)
N1—C3	1.312 (4)	C7—C8	1.420 (5)
N2—N3	1.299 (4)	C8—H8	0.9300
N2—C3	1.396 (4)	C8—C9	1.345 (5)
N3—C4	1.374 (4)	C9—H9	0.9300
N4—C7	1.344 (5)	C10—H10A	0.9600
N4—H4A	0.865 (10)	C10—H10B	0.9600
N4—H4B	0.868 (10)	C10—H10C	0.9600
C1—H1	0.9300		
			
C1—S1—C3	88.6 (2)	O1—C5—C4	115.8 (3)
C5—O1—C10	117.7 (3)	O1—C5—C6	123.9 (3)
H3A—O3—H3B	112 (5)	C6—C5—C4	120.2 (3)
C3—N1—C2	109.0 (3)	C5—C6—H6	119.6
N3—N2—C3	111.9 (3)	C5—C6—C7	120.8 (3)
N2—N3—C4	115.8 (3)	C7—C6—H6	119.6
C7—N4—H4A	120 (3)	N4—C7—C6	120.7 (4)
C7—N4—H4B	118 (3)	N4—C7—C8	120.2 (4)
H4A—N4—H4B	121 (4)	C6—C7—C8	119.1 (3)
S1—C1—H1	124.8	C7—C8—H8	119.9
C2—C1—S1	110.5 (3)	C9—C8—C7	120.2 (4)
C2—C1—H1	124.8	C9—C8—H8	119.9
N1—C2—H2	121.7	C4—C9—H9	119.0
C1—C2—N1	116.6 (3)	C8—C9—C4	122.1 (4)
C1—C2—H2	121.7	C8—C9—H9	119.0
N1—C3—S1	115.3 (3)	O1—C10—H10A	109.5
N1—C3—N2	120.4 (3)	O1—C10—H10B	109.5
N2—C3—S1	124.3 (3)	O1—C10—H10C	109.5
N3—C4—C5	117.5 (3)	H10A—C10—H10B	109.5
N3—C4—C9	124.9 (3)	H10A—C10—H10C	109.5
C5—C4—C9	117.6 (3)	H10B—C10—H10C	109.5
			
S1—C1—C2—N1	0.2 (5)	C3—S1—C1—C2	−0.2 (3)
O1—C5—C6—C7	179.3 (3)	C3—N1—C2—C1	−0.1 (5)
N2—N3—C4—C5	174.2 (3)	C3—N2—N3—C4	−177.8 (3)
N2—N3—C4—C9	−3.2 (5)	C4—C5—C6—C7	−0.8 (5)
N3—N2—C3—S1	2.3 (4)	C5—C4—C9—C8	−0.2 (5)
N3—N2—C3—N1	−178.1 (3)	C5—C6—C7—N4	−178.9 (3)
N3—C4—C5—O1	2.8 (5)	C5—C6—C7—C8	0.8 (5)
N3—C4—C5—C6	−177.0 (3)	C6—C7—C8—C9	−0.5 (6)
N3—C4—C9—C8	177.1 (3)	C7—C8—C9—C4	0.2 (6)
N4—C7—C8—C9	179.2 (4)	C9—C4—C5—O1	−179.6 (3)
C1—S1—C3—N1	0.2 (3)	C9—C4—C5—C6	0.5 (5)
C1—S1—C3—N2	179.7 (3)	C10—O1—C5—C4	−171.3 (3)
C2—N1—C3—S1	−0.1 (4)	C10—O1—C5—C6	8.6 (5)
C2—N1—C3—N2	−179.7 (3)		

### Hydrogen-bond geometry (Å, º)

**Table:**

*D*—H···*A*	*D*—H	H···*A*	*D*···*A*	*D*—H···*A*
N4—H4*A*···N2^i^	0.87 (1)	2.30 (2)	3.137 (5)	162 (3)
N4—H4*B*···O3	0.87 (1)	2.08 (1)	2.946 (5)	173 (4)
O3—H3*A*···N1^ii^	0.84 (1)	2.12 (2)	2.954 (5)	169 (6)
O3—H3*B*···N3^iii^	0.84 (1)	2.43 (3)	3.186 (5)	150 (5)
C9—H9···N4^iv^	0.93	2.69	3.587 (5)	162
C2—H2···N2^v^	0.93	2.87	3.798 (5)	176
C8—H8···O1^vi^	0.93	2.72	3.452 (5)	136
C1—H1···O3^vii^	0.93	2.56	3.463 (5)	165
C10—H10*C*···C3^ii^	0.96	2.89	3.655 (5)	137
C10—H10*A*···C6^iii^	0.96	2.83	3.551 (5)	132
C10—H10*A*···C7^iii^	0.96	2.86	3.502 (5)	125

Symmetry codes: (i) −*x*+3/2, *y*−1/2, −*z*+1/2; (ii) *x*+1/2, −*y*+3/2, *z*+1/2; (iii) −*x*+1, −*y*+1, −*z*+1; (iv) −*x*+3/2, *y*+1/2, −*z*+1/2; (v) −*x*+1/2, *y*+1/2, −*z*+1/2; (vi) *x*+1/2, −*y*+3/2, *z*−1/2; (vii) *x*−1, *y*+1, *z*.
